# Feasibility of membrane ultrafiltration as a single-step clarification and fractionation of microalgal protein hydrolysates

**DOI:** 10.3389/fbioe.2022.957268

**Published:** 2022-08-30

**Authors:** Laura Soto-Sierra, Zivko L. Nikolov

**Affiliations:** ^1^ Molecular Templates, Inc. (MTEM), Austin, TX, United States; ^2^ Texas A&M University, College Station, TX, United States

**Keywords:** microalgae, biorefinery, protein, membrane ultrafiltration, depth filtration

## Abstract

Protein hydrolysates are one of the most valuable products that can be obtained from lipid-extracted microalgae (LEA). The advantages of protein hydrolysates over other protein products encompass enhanced solubility, digestibility, and potential bioactivity. The development of an economically feasible process to produce protein hydrolysates depends on maximizing the recovery of hydrolyzed native protein from the lipid-extracted algal biomass and subsequent fractionation of hydrolyzed protein slurry. Previously, we reported a method for fractionation of enzymatically generated protein hydrolysates by acidic precipitation of algal cell debris and unhydrolyzed protein, precipitate wash, centrifugation, and depth filtration. The present study evaluates tangential flow ultrafiltration as a single-step alternative to centrifugation, precipitate wash, and depth filtration. The results demonstrate that the tangential flow ultrafiltration process has a potential that deserves further investigation. First, the membrane diafiltration process uses a single and easily scalable unit operation (tangential flow filtration) to separate and “wash out” hydrolyzed protein from the algal residue. Second, the protein recovery yield achieved with the tangential flow process was >70% compared to 64% previously achieved by centrifugation and depth filtration methods. Finally, protein hydrolysates obtained by membrane ultrafiltration exhibited slightly better heat and pH stability.

## Introduction

Enzymatic hydrolysis of protein-rich feedstocks has emerged as a versatile method for enhancing protein extractability of complex proteins and increasing the value of protein products. The available data from aqueous extraction of protein-rich meals (soy, rapeseed, and microalgae) indicate that enzyme-assisted protein extraction could significantly improve protein extractability and generate a variety of partially hydrolyzed products (Morris et al., 2008; Safi et al., 2017; Soto-Sierra et al., 2018; López-Pedrouso et al., 2020). Among potential protein products that could be generated from protein-rich feedstocks, including microalgae, protein hydrolysates are of a particular interest as their thermal and acidic pH stability makes them better suited as protein supplements in sports and nutritional drinks than protein isolates (Olsen and Adler-Nissen, 1979; Adler-Nissen, 1986). Lipid-extracted microalgae (LEA) is a particularly attractive feedstock for protein products because the solvent extraction of high-value lipids such as omega fatty acids (FA) and lutein (Kulkarni and Nikolov, 2017; Soto-Sierra et al., 2020) increases the protein content in extracted biomass residue (% dw) and reduces protein-rich biomass cost ($/kg) as much as an order of magnitude (Soto-Sierra et al., 2020).

Recent studies revealed that *1*) the microalgal cell wall was a barrier to protein (enzyme) hydrolysis and *2*) cell wall disruption/lysis improves enzyme-assisted protein extraction yields by as much as 50% (Safi et al., 2017; Akaberi et al., 2019; Soto-Sierra et al., 2021). Maximizing protein hydrolysis and release of the hydrolyzed protein in the aqueous extract slurry is the first step in the production of hydrolysates followed by recovery and purification of the hydrolyzed protein slurry. To ensure required product purity and stability for specialty food and drink applications, lysed algal residue and nonprotein impurities (chlorophyll pigments and starch) must be efficiently removed from the protein hydrolysates (Soto-Sierra et al., 2021). The removal of undesirable algal residue and soluble impurities can be achieved by a combination of downstream processing methods such as precipitation (Morris et al., 2008; Safi et al., 2017), centrifugation (Schwenzfeier et al., 2011), dead-end filtration (depth filtration), and tangential flow filtration (TFF) (Safi et al., 2014; Kulkarni and Nikolov, 2017; Safi et al., 2017). The selection and sequence of process unit operations depend on target protein molecular weight (MW) and solubility, particle size of lysed lipid-extracted biomass, and product yield and purity.

We recently compared several bench-scale options for preparation of protein hydrolysates from intact (unbroken) LEA, lysed LEA, and algal protein concentrates (Soto-Sierra et al., 2021). Based on the rate for enzymatic hydrolysis, protein yield, and production cost, direct proteolysis of lysed LEA emerged as the best starting material for the preparation of protein hydrolysates. The direct hydrolysis process of lysed LEA consisted of acidic precipitation of insoluble impurities (cell debris and unhydrolyzed protein complex including pigments) followed by centrifugation and depth filtration (Soto-Sierra et al., 2021). To maximize the yield of hydrolyzed proteins, the precipitated material was centrifuged, washed with water to release trapped peptides, and then again centrifuged. Combined supernatants were clarified by depth filtration to yield a hydrolysate that was free of chlorophyll pigments. The hydrolysate contained 63% protein, and protein recovery was about 64%. We determined that the protein content of hydrolysates could be increased to 73% by including an ion-exchange demineralization step. Although the latter step slightly reduced the protein hydrolysate yield, we believe that ion-exchange demineralization would add a significant toll to the product cost. The aforementioned process uses off-the-shelf equipment and reduces the presence of chlorophyll pigments in the final product but delivers an underperforming process yield of 64%. The need for a precipitate wash and extra centrifugation step to release trapped protein hydrolysate molecules led us to consider TFF as a process alternative in place of centrifugation, washing, and depth filtration steps.

In this study, we compare the yield and quality of protein hydrolysates produced on a bench scale by TFF ultrafiltration and the process described previously (centrifugation, wash, and depth-filtration) without the ion-exchange demineralization step. The criteria for evaluating the quality of hydrolysates obtained by each process consisted of heat and pH stability of aqueous hydrolysate samples and discoloration of freeze-dried hydrolysates upon heating.

## Methods

### Preparation of protein hydrolysate

#### Lipid extraction

Lipid-extracted algae (LEA) was generated by a previously developed protocol (Soto-Sierra et al., 2021). Frozen *Nannochloropsis sp* biomass (donated by Qualitas Inc.) was first thawed at room temperature and then extracted in 50 ml EtOH/g-DW biomass in two steps at 60°C. Biomass extraction and re-extraction were performed under the same conditions with an incubation time of 45 min/step. An extraction temperature of 60°C was selected to maximize solubility and extractability of carotenoids, chlorophyll, and lipids in EtOH. At the end of the process, the dry base composition of LEA was approximately 40% protein, 19% ash, 10% lipids, and 16% carbohydrates.

### LEA disruption and proteolysis

LEA slurry (10% solids) was milled at pH 11 using a 0.4 L High Energy Planetary Ball Mill System from MSE Supplies (Tucson, AZ, United States). The ball mill chamber was filled with 0.5 mm diameter zirconia beads (50% by volume) as per the manufacturer’s recommendation. To maximize cell disruption, ball milling time was set to 120 min.

The pH of the lysed LEA slurry was brought to pH 9.5 with 1 M HCl and heated to 50°C under continuous mixing to maximize enzyme activity. The protein hydrolysis reaction was started by adding Alcalase liquid preparation with a specific activity of <0.75 Anson units/ml (Calbiochem^®^) three doses at preselected concentrations of 0.9, 1.8, and 3.5% v/w-protein. During the hydrolysis reaction (3 h), the pH was maintained at 9.5 by the addition of 1 M NaOH. At the end of the reaction, the LEA hydrolysate slurry was acidified with 1 M HCl to pH 4.5 and then incubated at 95°C for 5 min to inactivate the enzyme. The hydrolyzed and acidified LEA slurry was cooled down to room temperature and then clarified by the two methods described in the following sections to obtain clarified (solid-free) protein hydrolysates.

### Process for the production of protein hydrolysates by centrifugation and depth filtration (DpF-hydrolysate)

**FIGURE 1:**
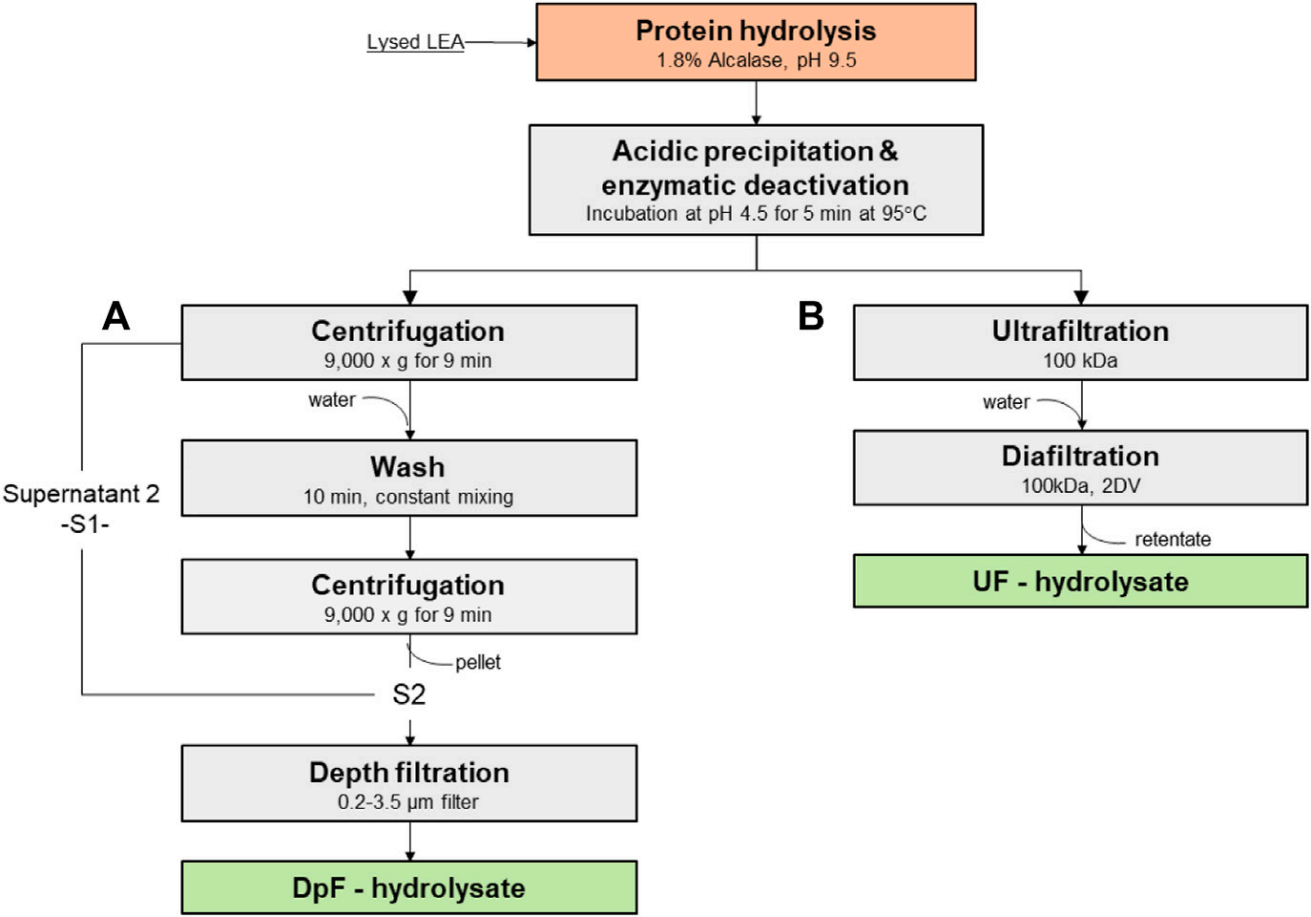
Processing routes to protein hydrolysates: **(A)** protein hydrolysates by centrifugation and depth filtration (DpF-hydrolysate) vs. **(B)** protein hydrolysates by ultrafiltration and diafiltration (UF-hydrolysate).

A modified protocol for clarification of protein hydrolyates developed by Soto-Sierra et al. (2021) was used (Figure 1A). After hydrolysis and deactivation, the slurry was centrifuged at 9,000 × *g* for 9 min, and the supernatant (S1) was collected. The pellet solids were resuspended in water at pH 4.3 and mixed thoroughly to release hydrolyzed protein trapped in the pellet. The resuspended pellet was centrifuged again under same conditions, and the supernatant S2 was collected. Supernatants S1 and S2 were combined and clarified using a Supracap 50 Pall^®^ Depth filtration capsule (SC050PDD1). The Supracap 50 Pall depth filter with a retention rating of 0.2–3.5 µm allowed the removal of residual debris and insoluble protein aggregates.

### Protein hydrolysates by ultrafiltration and diafiltration (UF-hydrolysate)

TFF and DF were performed using the Spectrum KrosFlo KR2i system with 1 mm inner diameter (ID) hollow fibers (Repligen) (Figure 1B). For all the experiments conducted, the shear rate was maintained at 2,000^−1^ or a cross-flow flow-rate of 19 L/min/m^2^. The membrane filtration process was operated at constant TMP (7 psi) until the system pressure was dictated by the viscosity of the fluid, which organically increased the feed pressure. At that point, the concentration (ultrafiltration) was stopped when a TMP of 10 was reached. The flux, TMP, and weight of permeate were tracked over time using KrosFlo real-time data collection software by Repligen.

Based on previous results (Morris et al., 2008; Soto-Sierra et al., 2021) and the anticipated MW distribution of the hydrolysates, 50 kDa (D02-E050-10-N) and 100 kDa (D02-E100-10-N) Spectrum^®^ hollow fiber filters were selected to clarify and purify protein hydrolysates from hydrolyzed and acidified LEA slurry. After hydrolysis and enzyme deactivation steps, the LEA hydrolysate slurry was concentrated by ultrafiltration until the solids’ concentration in the retentate reached ∼200 g-DW/L, or the feed pressure exceeded 10 psi. Following the concentration of the slurry, the Spectrum^®^ hollow fiber system was operated under the continuous diafiltration mode for two diafiltration volumes (DVs). To determine protein recovery in the permeate, samples were taken before and after each diafiltration step (DV1 and DV2), and the protein yield was calculated at each point.

### Characterization of protein hydrolysates

The protein hydrolysate yield was estimated using a protocol for quantification of soluble peptides previously developed and modified by Olsen and Adler-Nissen, (1979) and Soto-Sierra et al. (2021). Each sample was digested in 6N HCl for 24 h until hydrolysis. Total amino nitrogen was determined using the nitrogen O-phthaldialdehyde (NOPA) procedure (Cuchiaro and Laurens, 2019), and the protein content in the samples by applying an amino-nitrogen-to-protein conversion factor of 6.25 (Soto-Sierra et al., 2021).

### Size exclusion chromatography

MW distribution of the hydrolyzed protein in hydrolysates was performed on an AKTA-purifier system using a TOSOH TSK gel G2000SWxl (30 cm × 7.8 mm) size exclusion analytical column with a TOSOH SWXL guard precolumn. All samples were filtered through a 0.2-µm filter before injection. Protein samples (100 µl) were run at a 0.7 ml/min flow rate using 0.1 M NaCl in RO water as the mobile phase. The protein in the effluent was detected using a UV detector at 280 nm. The retention volumes of a standard protein mixture (Bio-Rad) were used to assign MW to protein hydrolysate peaks.

### Analysis of pH stability of hydrolysates at an elevated temperature

Hydrolysate samples (10 mg/ml) prepared by either fractionation methods were adjusted to pH 4, 6, and 8. Only a half of the pH-adjusted samples were subjected to thermal treatment at 95°C for 10 min. A volume of 1 mL of each sample (heated and non-heated) was analyzed by dynamic light scattering (DLS) for particle size measurements in the Zetasizer Nano ZS at 25°C and 173° scattering angle. The DLS data were automatically converted to intensity and volume distribution by particle size.

### Data analysis

Statistical analysis of process variables was performed using JMP software. Significant differences between treatments, where applicable, were found using α_FAM_ = 0.05.

## Results and discussion

### Protein hydrolysate yield using the UF/DF process

**FIGURE 2:**
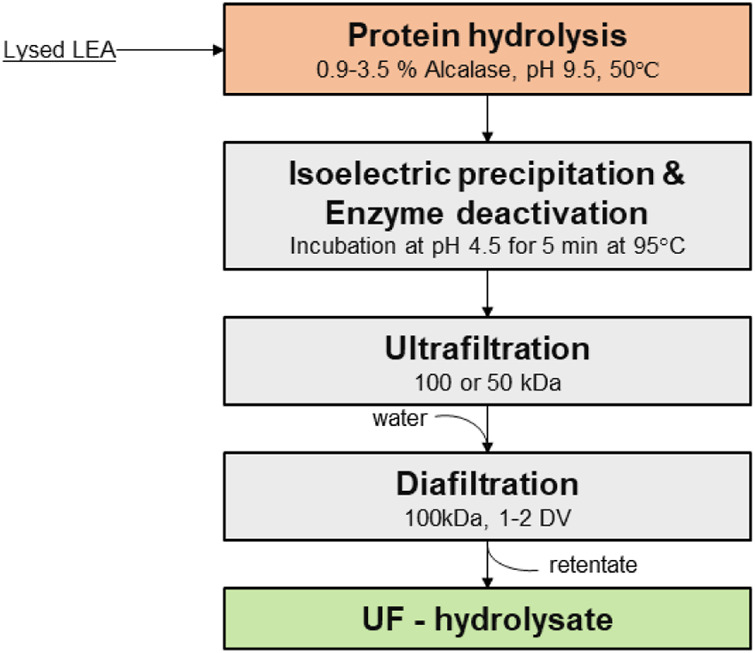
Flow diagram of the process for the production of UF-hydrolysates.

To determine conditions for the separation of protein hydrolysate from the rest of the components in the lysate slurry, four key process variables were evaluated: enzyme dosage (0.9, 1.8, and 3.5% w/v), pH (4.5, 5.5, and 7.0), enzyme deactivation temperature (45 and 60°C), and membrane pore size (50 and 100 kDa). The objective of the screening was to determine the optimal extent of hydrolysis and ultrafiltration conditions that allowed maximal passage of hydrolyzed protein while retaining insoluble protein aggregates, chloroplast remnants, and cell debris. From the statistical analysis, we found that enzyme dosage, membrane pore size (MWCO), and their interaction were the only significant factors affecting protein recovery and flux. The effect of these factors was evaluated by testing protein recovery and flux at the conditions shown in Figure 2.

Hydrolysates produced with 0.9% and 1.8% (v/w) enzyme dosages and processed *via* a 50 kDa hollow fiber membrane delivered lower fluxes and cumulative protein yield than those produced by the other three tested combinations (Table 1). The combination of 0.9% dosage and 50-kDa membrane had the lowest flux (16 LMH) and protein yields during UF concentration (33%) and subsequent two diafiltration steps (48% and 56%, respectively).

**TABLE 1:** Impact of the MWCO membrane and enzyme dosage on flux and protein recovery in the permeate and after one and two diafiltration volume (DV).

Cumulative permeate protein yield					
Alcalase dosage (%v/w)	MWCO membrane (kDa)	Average flux (LMH)	UF (%w/w)	DV1 (%w/w)	DV2 (%w/w)
3.5	100	25 ± 2	50 ± 5	63 ± 4	73 ± 2
1.8	100	25 ± 1	53 ± 5	65 ± 4	73 ± 3
3.5	50	32 ± 6	46 ± 4	61 ± 4	70 ± 3
1.8	50	20 ± 0	41 ± 5	57 ± 2	66 ± 2
0.9	50	16 ± 3	33 ± 7	48 ± 6	56 ± 6

The increase of Alcalase dosage from 0.9 to 1.8 or 3.5% (v/w) resulted in higher permeate fluxes of 20 and 32 LMH, respectively, when processing the hydrolyzed slurry using a 50-kDa membrane. Similarly, the same dosage increase from 0.9 to 1.8% and 3.5% positively affected the UF permeate protein yield (41 and 46%, respectively) and the cumulative yield after the second diafiltration (second DV) step (66% and 70%, respectively) (Table 1).

Interestingly, 100 kDa ultrafiltration and diafiltration (UFDF) of protein hydrolysates that were generated with either 1.8% or 3.5% enzyme dosage did not result in significantly different fluxes or protein yields. The UFDF process of the hydrolyzed slurry at 3.5% Alcalase had a higher permeate flux when processed by 50-kDa rather than 100-kDa hollow fiber membrane, but the protein yield was not substantially different.

**FIGURE 3:**
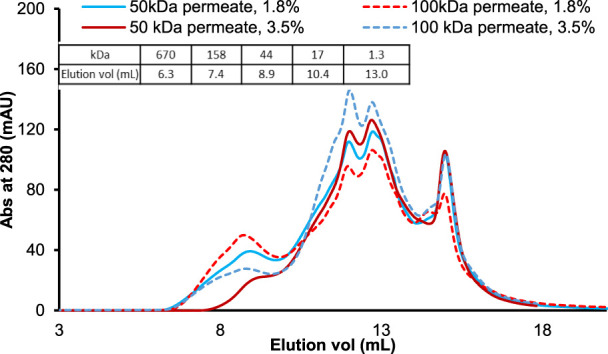
MW distribution of UF permeates obtained using 50-kDa (solid line) and 100-kDa (dotted line) hollow fiber membranes and 1.8% v/w and 3.5% v/w enzyme dosages. The chromatograms of UF permeate samples were generated on a TSK gel G2000swxl column (30 × 0.7 cm) at a flow rate of 0.7 ml/min. Bio-Rad protein standard mix ranging from 1.3 to 670 kDa was used to assign the estimated MW of hydrolysate peaks (*see* table inset).

To examine further flux and protein yield data, we then investigated the size-exclusion chromatography (SEC) profiles of protein hydrolysates produced with two different enzyme dosages and membrane pore sizes (Figure 3). The hydrolysate MW profiles in Figure 3 were obtained using UF permeates rather than diafiltrates to obtain a stronger UV (280 nm) detector response and detect low concentration protein species. The comparison of the protein elution profiles of hydrolysates produced with 1.8% and 3.5% Alcalase dosages shows that higher enzyme dosage reduces the amount of higher MW protein fragments that elute between 7 and 10 ml elution volumes. The decrease of larger MW fragments is reflected by an increase in lower MW peaks that elute between 11 and 13 ml. Based on the protein standard curve, protein elution volumes of 7–10 ml translate to approximately 158–17 kDa MW range and 11–13 ml to 5–2 kDa. Therefore, one would expect the higher enzyme dosage (3.5%) to impact the performance more of the tighter 50-kDa membrane than the 100-kDa one (Table 1). The data in Table 1 show that the UF permeate flux with the 50-kDa MWCO hollow-fiber membrane increased from 20 to 32 LMH, but the flux through the 100-kDa membrane did not change with the enzyme dosage (25 LMH). Similarly, UF permeate yields were notably affected by the enzyme dosage when processing hydrolysates through the 50-kDa membrane (an increase from 41 to 46%) than 100-kDa membrane (similar ∼50% yield).

To increase hydrolysate product yield in the permeate fraction, we implemented a diafiltration step for the recovery of additional hydrolyzed protein that had remained in the retentate at the end of the UF concentration step.

Diafiltration was performed in two stages each with one diafiltration volume exchange. Each diafiltration volume exchange protein yield was calculated, added to the previous step (UF or first DV), and reported as the cumulative protein yield (Table 1). The diafiltration results summarized in Table 1 show cumulative protein yields as a function of membrane pore size and enzyme dosage. The data indicate that diafiltration of UF or first DV retentate increases protein yield by about 10% and that after two DV exchanges one could achieve a cumulative protein yield greater than 70% with a 100-kDa MWCO hollow fiber membrane. The largest jump in protein recovery was observed with the 50-kDa membrane. After the first DV step (first DV), the protein yield was 15% higher followed by additional 10% after the second diafiltration step (second DV), irrespective of the enzyme dosage. As pointed out previously, the separation efficiency of the 50-kDa membrane was affected by the enzyme dosage and the cumulative protein yield after the second DV could reach 70% only for hydrolysates produced using 3.5% (v/w) Alcalase.

In summary, hydrolysates produced with 3.5% provide greater processing flexibility and, if the enzyme cost does not contribute significantly to the final product cost, one should consider using 3.5% (v/w) irrespective of the MWCO membrane (i.e., 50 or 100 kDa). Otherwise, the use of 1.8% (v/w) dosage would limit LEA hydrolysate processing (UFDF) to a 100-kDa hollow-fiber membrane. Because the lower enzyme dosage of 1.8% (v/w) and 100-kDa MWCO were an effective combination for enhancing the ultrafiltration flux and cumulative protein recovery yield, we selected the latter combination to produce membrane-processed hydrolysates (UF-hydrolysates). From this point forward, we aim to characterize and compare UF-hydrolysates alongside hydrolysates processed by centrifugation and depth filtration (DpF-hydrolyzates), following a process developed in one of our previous studies (Soto-Sierra et al., 2021). The main objective was to understand the advantages and disadvantages of single-step UFDF vs. centrifugation followed by depth filtration in terms of quality of the final hydrolysates. The exact process steps and key process parameters for production UF- and DpF-hydrolyses are depicted in Figure 1.

### Temperature (T) and pH stability of depth-filtered (DpF) and ultrafiltered (UF) hydrolysates

**FIGURE 4:**
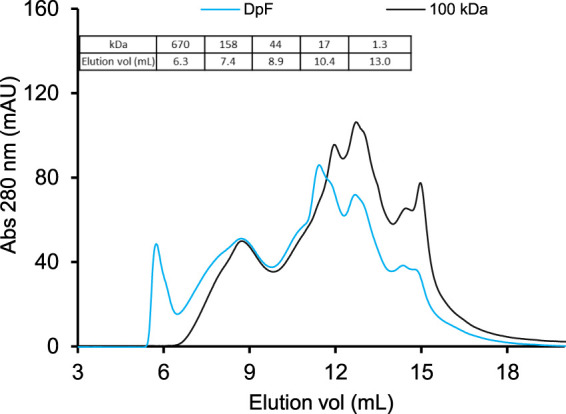
Size-exclusion chromatograms of DpF-hydrolysates (blue line) and UF-hydrolysates (black line) using 1.8% enzyme dosage. The chromatograms were generated on a TSK gel G2000swxl column (30 × 0.7 cm) at a flow rate of 0.7 ml/min. Bio-Rad protein standard mix was used to assign the estimated MW of hydrolysate peaks (table inset).

The temperature (T) and pH stability of hydrolysates are important quality attributes as these two parameters can contribute to product discoloration and reduced solubility due to protein aggregation (Lan et al., 2018; Edwards and Jameson, 2020; He et al., 2022). Because the stability of protein hydrolysates is known to be affected by the protein MW and composition (Adler-Nissen et al., 1978; Olsen and Adler-Nissen, 1979), we first determined SEC elution profiles of UF-hydrolysates and DpF-hydrolysates (Figure 4)

The MW distribution and peak intensities of the two hydrolysates were similar except for the eluting protein in 5–7 ml and absorption intensity of protein peaks at 12 and 15 ml.

The molecular weight profiles in Figure 4 show that the DpF-hydrolysate (blue elution profile in Figure 4) contained a fraction of high-MW protein (>670 kDa) that eluted in ∼6 ml. The latter protein fraction was not present in the UF-processed samples because 100-kDa MWCO membranes are intended to reject most protein molecules greater than 300 kDa size. The data indicate that centrifugation followed by a depth filtration process produces DpF-hydrolysates with a broader MW range than the UF process. This observation is consistent with limited fractionation power of acidic precipitation, which is governed by protein physicochemical properties such as solubility, hydrophobicity, and net charge at the precipitation pH (Bramaud et al., 1997) rather than their MW size as it is the case with membrane separations.

The 100-kDa membrane-processed hydrolysates (black line) shown in Figure 4 consist mostly of protein fractions ranging from 44 to 1.3 kDa that have eluted between 9 and 15 ml elution volumes. Protein peaks at about 15 ml in both samples correspond to an estimated MW of less than 1 kDa and consist of dipeptides, tripeptides, and free amino acids. The question that remains is whether the slight difference in protein composition would result in detectable stability differences. Specifically, would the absence of the largest MW protein fraction (5–7 ml elution volume) in the UF-processed LEA hydrolysates increase pH and heat stability of the UF-hydrolysates compared to DpF-hydrolysates?

**FIGURE 5:**
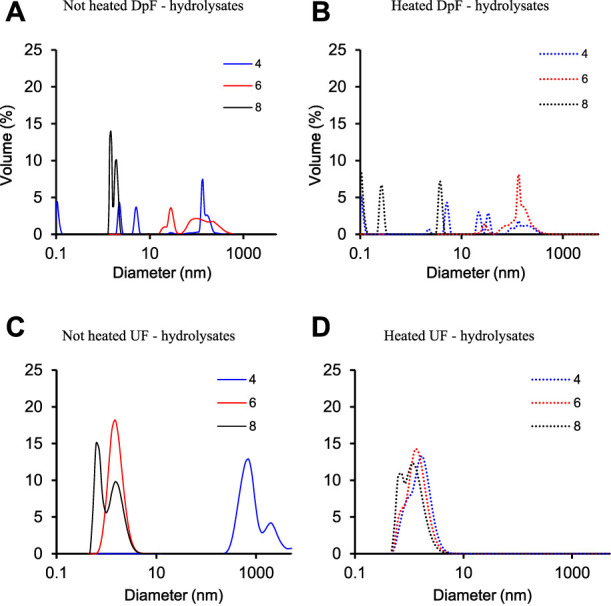
Molecular size distribution of **(A)** non-heated DpF-hydrolysates; **(B)** heated DpF-hydrolysates; **(C)** non-heated UF-hydrolysates; **(D)** heated UF-hydrolysates at pH 4, 6, and 8.

To address this question, the molecular (particle) size distribution of heated and non-heated DpF- and UF-hydrolysate samples was compared at three different pHs (4, 6, and 8) using dynamic light scattering (DLS). The molecular weight size distribution of heated and non-heated DpF- and UF-hydrolysate samples as a function of pH at 95°C is summarized in Figure 5.

The results in Figure 5 show that the size distribution of UF and DpF protein hydrolysates was different for both heated and non-heated samples. A significant size distribution shift in DpF-hydrolysates was observed upon pH adjustment and heating (Figures 5A,B). The DLS graphs of heated and non-heated samples at pH 4.0 (blue line) and 6.0 (red line) show peaks between 100 and 1000 nm, which are an indication of aggregation (Filipe et al., 2010). At pH 8.0 (black solid line), the size distribution changed slightly upon heating but remained between 0.1 and 10 nm.

The UF-hydrolysates (Figures 5C,D), on the other hand, were more stable at the three pHs before and after heating as evidenced by the comparable size distribution profiles. The volumetric fraction diameter of UF-processed LEA-hydrolysates ranged from 0.2 to 10 nm in all the treatments but in the non-heated samples at pH 4.0. The non-heated samples at pH 4.0 (blue solid line) resulted in two peaks with volumetric fraction diameters around 1000 nm. We hypothesize that pH 4.0, which is very close to the average pI of algae proteins (pH 4.0–5.5) (Ursu et al., 2014), induced the association of protein fragments in the hydrolysate that led to the observed shift of the volumetric faction diameter (Goudarzi et al., 2015). The absence of later peaks upon heating of the same samples suggests that thermal energy (95°C) might have reduced/disrupted molecular interactions causing the presumed protein association (Goudarzi et al., 2015). Based on DLS data and SEC profiles, physicochemical properties of UF-hydrolysates would be more predictable and probably a better choice for the development of food and drink formulations.

**FIGURE 6:**
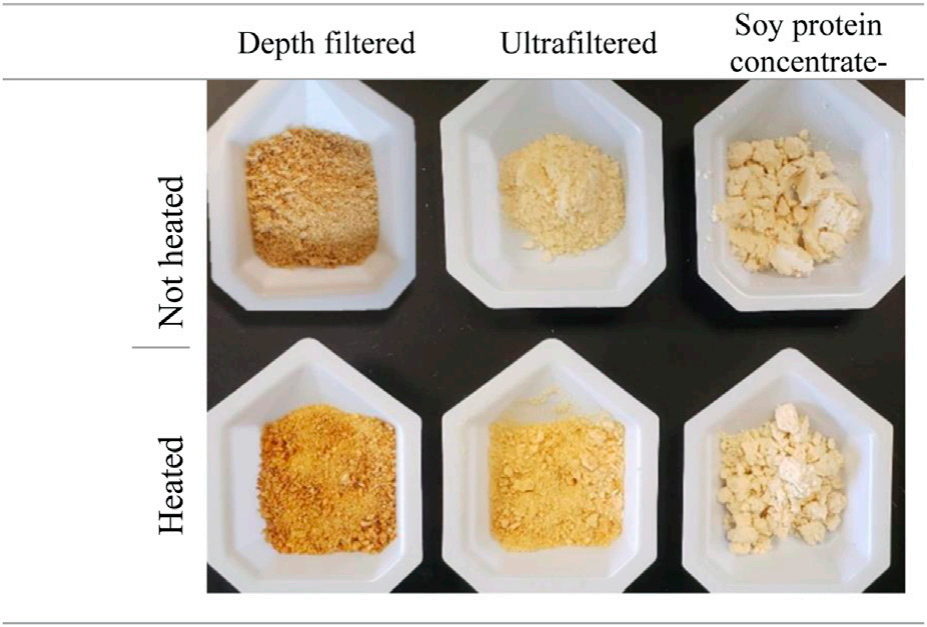
Effect of heating (95°C for 10 min) on depth-filtered (DpF-hydrolysates) and ultra-filtered (UF-hydrolysates) hydrolysates. –Non-heated samples are shown in the top row and heated ones on the bottom.

To further evaluate the impact of heating on the hydrolyzed samples, we compared the browning reaction of freeze-dried DpF-hydrolysates and UF-hydrolysates to a soy protein concentrate control (Figure 6). When the soy protein concentrate did not show any signs of browning, we observed some darkening of the UF-hydrolysate sample, and a significantly greater browning of the DpF sample due to Maillard reactions and potential degradation (Yu et al., 2018; Fu et al., 2020). The results of the browning reaction of the freeze-dried hydrolysates support the aforementioned conclusion that UF-hydrolysates are of superior quality. The higher quality of UF-hydrolysates compared to that of DpF-hydrolysates could be explained by the latter containing larger MW peptides (>10 kDa peptides), which are known to be more propense to degradation (Lan et al., 2010; Yu et al., 2018). The results suggest that the UF-hydrolysates would be a better candidate than DpF-hydrolysates in applications where pH and temperature stability matter.

## Conclusion

In this study, we evaluated a clarification process option for the production of algal protein hydrolysates using single-step ultrafiltration and diafiltration production of UF-hydrolysates. The results showed that the yields and MW distribution profile of protein hydrolysates were a function of the enzyme dosage during hydrolysis and the membrane (pore size) MWCO. The combination of higher dosage (3.5%) and 50-kDa MWCO membrane produced hydrolyzates with an overall lower molecular weight range, while lower enzyme dosage (0.9 and 1.8%) and 100-kDa MWCO resulted in hydrolysates with a higher number of proteins over 100 kDa. Protein hydrolysates that were generated with either 1.8% or 3.5% enzyme dosage and processed through a 100-kDa MWCO hollow fiber membrane had higher protein yields.

DpF-hydrolysates had a broader MW range and overall higher MW than the UF-hydrolysates. The presented data indicate that UF-hydrolysates would be more stable in pH and temperature and less susceptible to Maillard reactions, thereby probably being a better choice for the development of food and drink formulations.

In summary, this study suggests that the tangential flow ultrafiltration process is a viable process option to the traditional protein fraction method. Potential advantages of TFF include single-step clarification of algal hydrolysates, greater protein recovery yield (70 % vs. 64%), and apparently better pH and heat stability.

## Data Availability

The raw data supporting the conclusion of this article will be made available by the authors, without undue reservation.
